# Effect of spinal fusion on joint space narrowing of the hip: comparison among non-fusion, short fusion, and middle or long fusion

**DOI:** 10.1186/s10195-022-00682-3

**Published:** 2023-01-09

**Authors:** Taku Ukai, Hiroyuki Katoh, Katsuya Yokoyama, Masato Sato, Masahiko Watanabe

**Affiliations:** 1 Department of Orthopaedic Surgery, Surgical Science, Tokai University School of Medicine, 143 Shimokasuya, Isehara, Kanagawa 259-1193 Japan; 2 Department of Orthopaedic Surgery, Tokai University School of Medicine Oiso Hospital, 21-1 Gakkyo, Oiso, Kanagawa 259-0198 Japan

**Keywords:** Hip osteoarthritis, Spinal fusion, Spinal parameters, Joint space narrowing

## Abstract

**Background:**

Lumbar fusion corrects spinal deformities and improves spinal complications. Hip osteoarthritis (OA) is strongly correlated with spinal mobility, and joint space narrowing of the hip after spinal fusion has gained attention. This study aimed to elucidate the effect of spinal fusion on hip joint space narrowing.

**Materials and methods:**

We retrospectively examined 530 hips of 270 patients who underwent spinal surgery. All the patients underwent whole-spine radiography before and at the final follow-up. Patients were divided into three groups (N group: non-spinal fusion, S group: up to three interbody fusions, and L group: more than four interbody fusions). The rates of joint space narrowing, spinal parameters (sagittal vertical axis, thoracic kyphosis, lumbar lordosis, sacral slope, pelvic tilt, and pelvic incidence), and limb length discrepancy at the final follow-up were compared. A multilinear regression analysis was performed to identify the risk factors for the rate of joint space narrowing.

**Results:**

The rate of joint space narrowing was significantly higher in the L group than in the N and S groups (*P* < 0.001). No significant difference in the rate of joint space narrowing was observed between the N and S groups. Multiple linear regression analysis revealed that the number of fusion levels (*p* < 0.05) and follow-up period (*p* < 0.001) were independent risk factors for joint space narrowing. Spinal parameters at the final follow-up were not independent risk factors.

**Conclusions:**

Long spinal fusion (more than four levels) led to significantly greater joint space narrowing of the hip than short (up to three levels) or no fusion. Spinal alignment did not affect joint space narrowing of the hip. Surgeons should be aware that more than four interbody fusions may result in worse joint space narrowing of the hip.

**Level of evidence:**

IV, retrospective study

## Introduction

Multiple factors have been found to be associated with hip osteoarthritis (OA) [1, 2]. The correlation between spinal alignment and hip OA has received attention since hip–spine syndrome [3] was first reported, the correlation between spinal alignment and hip OA has received attention. Spinal alignment and pelvic inclination changes with aging, and these changes directly affect the hip joint loading. Spinal inclination and tilting affect the load on the hip joints [4, 5]. In addition, a large pelvic tilt (PT) is observed in patients with rapidly destructive coxarthrosis [6, 7]. A large pelvic incidence (PI), sacral slope (SS), and PI minus lumbar lordosis (LL) are associated with hip OA [8]. However, some reports showed that PI was not associated with hip OA [9], and so this relationship remains inconclusive [10]. Therefore, the correlation between spinal alignment and hip OA remains unclear.

Spinal fusion has been performed to fix spinal instability and correct spinal malalignment.

Spinal surgery widely contributes to reducing pain, such as that from myelopathy and hernia, and improves activities of daily living. However, there is a possibility that spinal fusion also affects joint space narrowing of the hip. Some studies reported that long spinal fusion accelerates joint space narrowing of the hip and increases the risk of total hip arthroplasty (THA) [11, 12]. Long spinal fusion was performed to correct sagittal malalignment. Although spinal alignment is improved by spinal fusion, spinal fusion may increase the mechanical load on the adjacent joints. Adjacent segmental degeneration (ASD) after spinal fusion has been reported in 16.5% (5 years after spinal fusion) and 36.1% (10 years after spinal fusion) of patients [13]. Similarly, asymptomatic and symptomatic ASD were reported in 26.6% and 8.5% of patients, respectively [14]. Spinopelvic joints play a crucial role during sitting-to-standing and standing-to-sitting postures. Therefore, spinopelvic fusion restricts mobility and increases the mechanical force on the hip to compensate for the restriction of spinopelvic mobility. Some studies have shown the drawbacks of spinopelvic fusion. Spinopelvic fusion is associated with dislocation after THA due to contact with the femoral neck and the acetabular rim [15, 16]. Other studies showed that lumbosacral fusion affects sacroiliac joint pain [17, 18].

Spinal fusion not only improves spinal alignment, but it also increases the mechanical load on adjacent joints and accelerates joint space narrowing. However, this discrepancy has not yet been fully elucidated. The purpose of this retrospective study was to determine (1) if spinal fusion affects joint space narrowing of the hip; (2) if spinal alignment affects joint space narrowing of the hip; and (3) how non-fusion and short and long spinal fusion affect joint space narrowing.

## Materials and methods

### Patients

This retrospective study examined 530 hips of patients (252 hips of 127 males and 278 hips of 143 females) who underwent spinal surgery between May 2010 and May 2019. This study was approved by the Institutional Review Board of the authors (22R118). The inclusion criteria were as follows: (1) age over 50 years, (2) pre- and postoperative whole standing X-rays, and (3) a follow-up period of over 2 years. The exclusion criteria were as follows: (1) a previously operated-on limb, (2) patients with no hip joint space during preoperative radiography, (3) connective tissue disease, (4) the absence of a pre- or postoperative whole standing X-ray, and (5) a follow-up period of less than 2 years. The requirement for informed consent was waived because of the retrospective nature of the study. The patients were divided into three groups (N group with no fusion; S group with up to three interbody fusions; L group with more than four interbody fusions). The number of patients in each group was as follows: N group, 85 patients with 167 hips; S group, 114 patients with 225 hips; L group, 71 patients with 138 hips.

Demographic data for the three groups are shown in Table 1.

**Table 1:** Demographic data for the three groups

	N group (167 hips of 85 patients)	S group (225 hips of 114 patients)	L group (138 hips of 71 patients)	*P* value N – S	S - L	N - L
Age (years)	69 ± 8.9	70.4 ± 7.3	70.4 ± 8.2	0.412	1	0.296
Sex (male:female)	59:26	60:54	8:63			
BMI	24 ± 3.8	23.9 ± 3.7	22.8 ± 4.1	1	0.024*	0.026*
CE (degrees)	26.9 ± 7.9	27.9 ± 7.6	26.5 ± 8.1	0.56	0.3	1
Sharp (degrees)	41.2 ± 4	41 ± 4.1	42.3 ± 5.2	1	0.022*	0.141
*Limb length discrepancy (mm)*	*3.2 ± 3.4*	*3.1 ± 2.6*	*3.3 ± 2.5*	*1*	*1*	*1*
Sacral fusion	0 / 167	18 / 225	112/138			
Number of fusion levels	0	1.4 ± 0.6	10 ± 3.0	< 0.001*	< 0.001*	< 0.001*
Follow up period (months)	35.8 ± 11.9	43.5 ± 15.4	48.9 ± 22.3	< 0.001*	0.008*	< 0.001*

*SD* standard deviation, *BMI* body mass index, *CE* center edge

* Statistically significant difference

### Surgical indication

Spinal surgery without fusion was performed in patients with foraminal stenosis and without instability. Short fusions (up to three interbody fusions) were performed to decompress foraminal stenosis or correct segmental instability. Middle or long fusion (more than four interbody fusions) was performed to correct global sagittal malalignment.

### Radiographic assessment

**Fig. 1:**
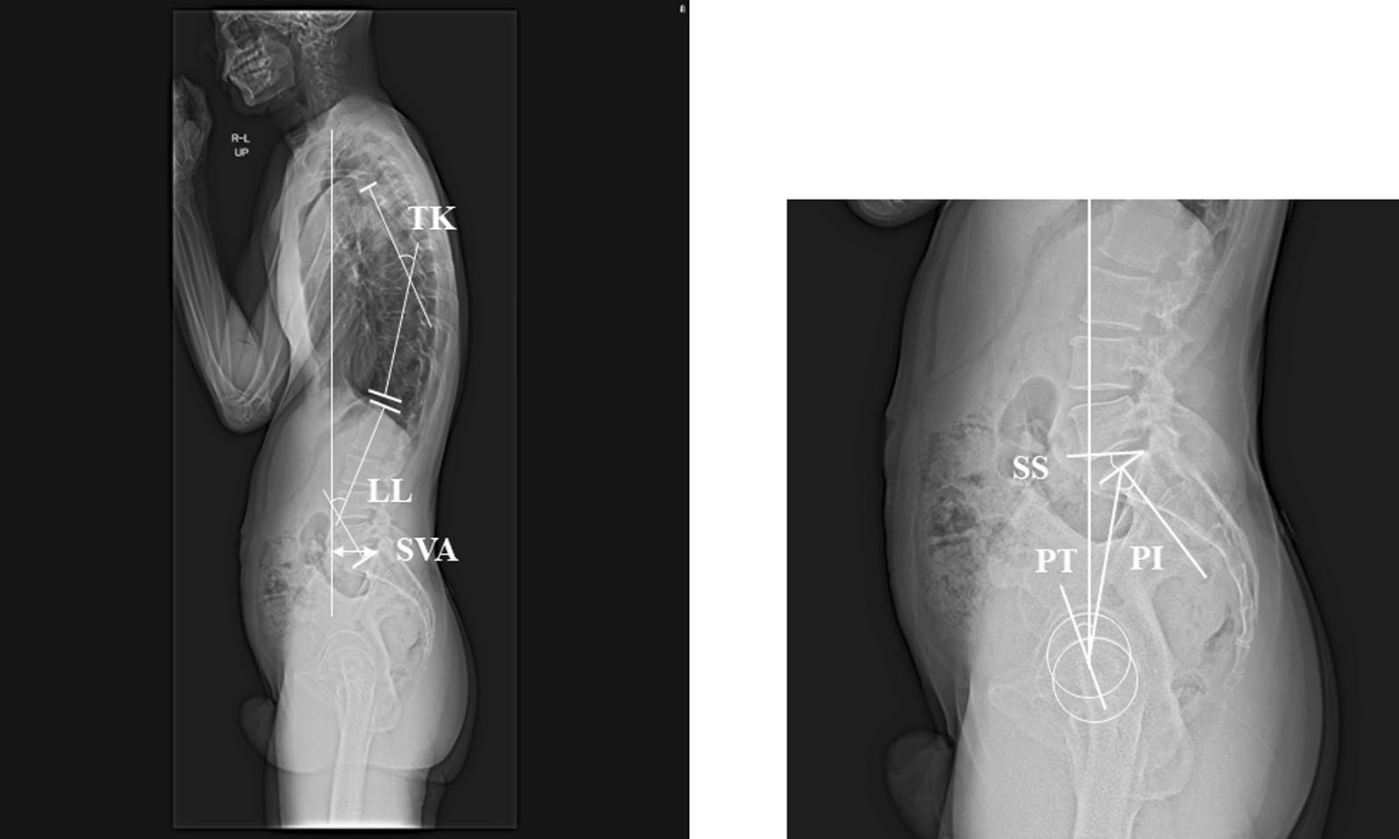
Measurements of spinal parameters. The SVA was defined as the distance between the vertical line from the center of the seventh vertebral body and the posterior edge of the first sacral vertebra. TK was defined as the angle between the parallel lines drawn along the twelfth inferior thoracic vertebra and the fourth superior thoracic vertebra. LL was defined as the angle between the parallel lines drawn along the first superior lumbar and sacral vertebrae. SS was defined as the angle between the horizontal line and first superior sacral vertebra. PT was defined as the angle between the vertical line and the line connecting the centers of the bilateral femoral heads. PI was defined as the angle between the vertical line from the superior first sacral vertebra and the line connecting the centers of the bilateral femoral heads.* SVA* sagittal vertical axis,* TK* thoracic kyphosis,* LL* lumbar lordosis,* SS* sacral slope,* PT* pelvic tilt,* PI* pelvic incidence

**Fig. 2:**
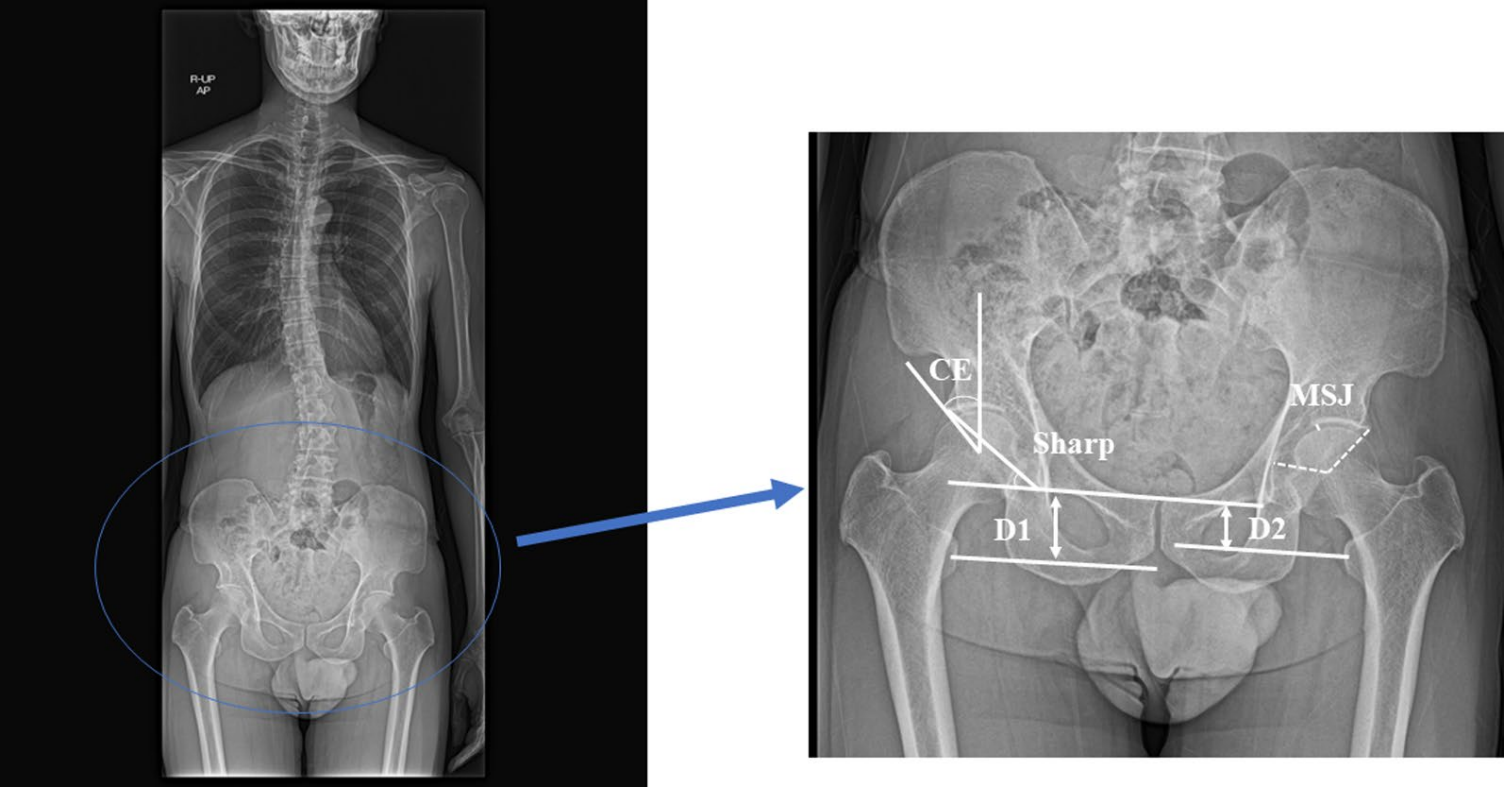
Measurements of hip parameters. The CE angle was defined as the angle between the vertical line and the lateral edge of the acetabulum. The Sharp angle was defined as the line connecting the bilateral teardrops and the lateral edge of the acetabulum. MJS was defined as the narrowest point from the lateral edge of the acetabulum and fovea. Limb length discrepancy was calculated as the perpendicular distance from the line connecting the bilateral teardrops to the center of each lesser trochanter. Limb length discrepancy was defined as the length of the right side (D1) minus that of the left side (D2) and recorded as an absolute value.* CE* center edge,* MJS* minimum joint space

Whole-spine standing radiography was performed preoperatively and more than 2 years after spinal surgery. Sagittal spine radiography was performed in the relaxed standing position. Patients were instructed to look ahead and place their hands on both clavicles [19]. The sagittal vertical axis (SVA), thoracic kyphosis (TK), lumbar lordosis (LL), SS, PT, and PI were evaluated as spinal parameters (Fig. 1) [20, 21]. The center edge (CE) angle [22], Sharp angle, limb length discrepancy [23], and minimum joint width (MJW) of the hip were evaluated as hip parameters (Fig. 2). MJW was measured in 0.1-mm increments between the lateral edge of the acetabulum and fovea (Fig. 2) [24]. The preoperative MJW and postoperative MJW were measured at the same point. The rate of joint space narrowing was calculated using the following formula: {preoperative MJW (mm) − postoperative MJW (mm)}/follow-up years [9].

All measurements were performed using a picture archiving and communication system (TechMatrix Corporation, Tokyo, Japan). Radiographic evaluation was performed by two orthopedic surgeons. A single surgeon evaluated all radiographic data, and the other orthopedic surgeon evaluated 80 randomly selected radiographic data values. Each surgeon performed the evaluation twice, and the average value was used for the evaluation. The intraclass reliability of the radiographic parameters was as follows: SVA (0.98); TK (0.8); LL (0.91); SS (0.94); PT (0.97); PI (0.92); CE (0.81); Sharp angle (0.83); limb length discrepancy (0.79); and MJW (0.8).

### Statistical analysis

Power analysis was performed to calculate the minimum sample sizes needed to perform linear multiple regression (effect size = 0.25, alpha = 0.05, power = 0.95, number of predictors = 15) and one-way analysis of variance (ANOVA) (effect size = 0.25, alpha = 0.05, power = 0.95, number of groups = 3). The calculated sample sizes were 125 and 252, respectively. The G-Power software (version 3.1.9.2, Germany) was used to calculate the sample size.

One-way ANOVA followed by the Bonferroni test were performed to compare the three groups. Multiple regression analyses were performed to identify the independent predictors of the rate of joint space narrowing. Independent variables were as follows: age, sex, body mass index, CE angle, Sharp angle, postoperative limb length discrepancy, postoperative SVA, postoperative TK, postoperative LL, postoperative SS, postoperative PT, postoperative PI, sacral fusion, number of lumbar fusion levels, and follow-up period. Statistical significance was set at a* P* value of < 0.05. SPSS software (version 26 IBM Corp., Armonk, NY, USA) was used to perform statistical analyses.

## Results

One-way ANOVA followed by Bonferroni revealed that the rate of joint space narrowing of the L group (0.10 ± 0.14 mm/year) was significantly higher than those of the other groups (N group; 0.07 ± 0.1 mm/year, S group; 0.06 ± 0.09 mm/year) (*P* < 0.001). Regarding the rate of joint space narrowing, no statistically significant difference was observed between the N and S groups. As for spinal parameters, the postoperative SVA of the L group (83 ± 63.7 mm) was significantly higher than that of the N group (52.9 ± 55.8 mm). The postoperative SVA of the S group (71.2 ± 53.2 mm) was significantly higher than that of the N group. The postoperative TK in the L group (45.4 ± 16.3°) was significantly higher than those in the other two groups (N: 31.8 ± 11.7°, S: 28.1 ± 12.2°). The postoperative LL of the S group (30.6 ± 15.8°) was significantly lower than those of the other two groups (L: 37.5 ± 18.5°, N: 36 ± 17.2°). The postoperative SS of the L group (23.4 ± 12.8°) was significantly lower than those of the other two groups (N: 28.4 ± 10.7°, S: 27 ± 11.1°). The postoperative PT of the N group (18.5 ± 10.5°) was significantly lower than those of the other two groups (L: 23.6 ± 11.6°, S: 22.7 ± 9.2°) (Table 2). Multiple regression analysis revealed that the number of fusion levels (*P* < 0.05) and follow-up period (*P* < 0.001) were independent risk factors for joint space narrowing (Table 3).

**Table 2:** Spinal parameters of each group at final follow-up

	N group	S group	L group	*P* value N - S		S - L	N - L
SVA (mm)	52.9 ± 55.8	71.2 ± 53.2	83 ± 63.7	0.005*		0.167	< 0.001*
TK (degrees)	31.8 ± 11.7	28.1 ± 12.2	45.4 ± 16.3	0.02*		< 0.001*	< 0.001*
LL (degrees)	36 ± 17.2	30.6 ± 15.8	37.5 ± 18.5	0.005*		0.001*	1
SS (degrees)	28.4 ± 10.7	27 ± 11.1	23.4 ± 12.8	0.684		0.012*	< 0.001*
PT (degrees)	18.5 ± 10.5	22.7 ± 9.2	23.6 ± 11.6	< 0.001*		1	< 0.001*
PI (degrees)	48.8 ± 11.3	51.2 ± 11.7	49 ± 13.1	0.156		0.291	1

*SD* standard deviation, *SVA* sagittal vertical axis, *TK* thoracic kyphosis, *LL* lumbar lordosis, *SS* sacral slope, *PT* pelvic tilt, *PI* pelvic incidence

* Statistically significant difference

**Table 3:** Results of linear multiple regression analyses

	Standard error	Standardized beta coefficient	*T* value	*P* value
Age (years)	0.001	− 0.019	− 0.404	0.687
Sex: female	0.011	− 0.059	− 1.145	0.253
BMI	0.001	0.045	1.030	0.304
CE (degrees)	0.001	− 0.078	− 1.456	0.146
Sharp (degrees)	0.001	0.06	1.086	0.278
*Limb length discrepancy (mm)*	*0.002*	*0.047*	*1.091*	*0.276*
SVA (mm)	0	− 0.016	− 0.241	0.81
TK (degrees)	0	− 0.038	− 0.618	0.537
LL (degrees)	0.001	0.006	0.054	0.957
SS (degrees)	0.002	0.002	− 0.109	0.914
PT (degrees)	0.002	− 0.069	− 0.387	0.699
PI (degrees)	0.002	− 0.029	− 0.138	0.89
Sacral fusion	0.019	− 0.048	− 0.647	0.518
Number of fusion levels	0.002	0.192	2.386	0.017*
Follow up period (months)	0	− 0.177	− 3.78	< 0.001*

*BMI* body mass index, *CE* center edge, *SVA* sagittal vertical axis, *TK* thoracic kyphosis, *LL* lumbar lordosis, *SS* sacral slope, *PT* pelvic tilt, *PI* pelvic incidence

* Statistically significant difference

## Discussion

This study revealed that the rate of joint space narrowing of the L group was significantly higher than those of the other two groups. No significant difference in the rate of joint space narrowing was observed between the N and S groups. The number of fusion levels and the follow-up period were independent risk factors for the rate of joint space narrowing.

The reported percentage of patients with adjacent segmental degeneration after spinal fusion ranges between 5% and 43% [13, 25–27]. This percentage is highly dependent on the follow-up period and the number of fusion levels. Some studies have reported the effects of long spinal fusion on joint space narrowing of the hip. Kawai et al. reported that more than seven spinal fusions accelerate joint space narrowing of the hip [11]. Other authors have reported that female patients with more than seven spinal fusions are more likely to develop THA [12]. However, these reports only included patients who underwent spinal fusion, and there are no reports that elucidate the effect of non-fusion and spinal fusion on joint space narrowing. Our results showed no significant difference in the rate of joint space narrowing between the N and S groups. This result indicates that a short fusion (less than three fusions) does not accelerate joint space narrowing of the hip, at least during a short follow-up period. In contrast, the L group had a higher rate of joint space narrowing than the other two groups. The mobility of the thoracolumbar spine in patients with hip OA was lower than that in healthy individuals [28]. In addition, lower spinal mobility has been reported to be a predictor of hip OA progression [4]. The mechanical overload of the hip increases after spinal fusion because hip motion is affected by spinal motion [29, 30], and restriction of the spinopelvic joint mobility compensates for excessive hip motion. This overload causes dislocation after THA [16] and may affect joint space narrowing of the hip. We consider that the hip overload was compensated for by the remaining spinal motion in short fusion and non-fusion. However, compensation does not work in long fusion because most of the lumbar or thoracolumbar joints are fixed. Hence, the rate of joint space narrowing increased only in the L group. Therefore, surgeons should pay attention to accelerated joint space narrowing of the hip, especially after a long fusion.

Limb length discrepancy is observed in hip OA as well as scoliosis patients [31, 32]. It may increase the load on the ipsilateral or contralateral limb and be associated with joint space narrowing of the hip. However, we found no significant differences in our ANOVA and multiple regression analysis. Therefore, we believe that limb length discrepancy does not affect short-term joint space narrowing of the hip.

Some authors have reported that spinal parameters affect the load on the hip joints and hip OA [8]. It has been reported that spinal anterior inclination and a larger SVA increase the load on the hip joint [4]. The load axis may shift anteriorly as SVA increases. Spinal anterior inclination can increase the internal hip extension moment and mechanical load of the hip [4]. The SVA of the L group was higher than that of the N group, but there was no significant difference between the two groups. From this result, we consider that the SVA may not affect the acceleration of the rate of joint space narrowing. LL has also been correlated with hip OA [33]. Pelvic retroversion occurs following lumbar spondylolisthesis and may progress to joint space narrowing of the hip. In this study, the LL in the N and L groups was larger than that in the S group because most of the N group patients did not have severe spinal deformity and the S group patients could not be corrected for sagittal alignment. However, the rate of joint space narrowing in the S group was not significantly different from that in the N group. Thus, other factors may be more affected than LL. PT is consistent with PI, and a higher PI is associated with hip OA. PI is a peculiar pelvic anatomy that remains unchanged even after 10 years [34, 35], and PI, SS, and PT change simultaneously. As PT increases, SS conversely decreases and the pelvis tilts posteriorly. Pelvic posterior tilt is observed in the aging population and is correlated with rapidly destructive coxarthrosis [36]. These spinal parameters may accelerate the rate of narrowing of the joint space. However, these parameters were not independent risk factors for joint space narrowing. Although spinal parameters were different among the three groups, we considered that the spinal fusion level affected the rate of joint space narrowing more than the spinal parameters.

This study has several limitations. First, this was a retrospective study, and several factors affected hip OA. Thus, we could not eliminate all the confounding factors. However, we calculated the sample size and analyzed adequate samples. In addition, we performed linear multiple regression analysis to eliminate the effects of confounding factors. Second, joint space narrowing progresses over a long period, and our average follow-up period was 2.98 years. Thus, a longer follow-up period is needed to elucidate a more detailed analysis. Third, we could not evaluate hip pain because of the retrospective study design. Hip pain is a chief symptom of hip OA; however, the precise association between hip OA and pain remains unclear [37–40], and most individuals with hip pain do not have radiographic hip OA [38, 41]. Thus, we believe that the evaluation of hip pain is not relevant to this study. Fourth, several methods have been used for the diagnosis of OA. The Kellgren–Laurence grade is commonly used with X-rays for hip OA [42–44]. However, there are only four possible grades, and joint space narrowing progresses gradually. Hence, it is difficult to evaluate slight changes in Kellgren–Lawrence grade. We measured MJS directly. This method has already been used to evaluate the rate of joint space narrowing [8, 11, 45], and our intraclass reliability was high. Sixth, we only measured joint space narrowing in this study, even though other findings, such as cartilage thickness, intra-articular fluid, and bone signal intensity changes, are also important. Thus, we plan to evaluate the correlation between spine fusion and these findings in the next study.

In conclusion, the rate of joint space narrowing after long fusions (more than four levels) was significantly higher than that after short fusions (up to three levels) and no fusion. Although spinal parameters were different among the three groups, they were not risk factors for acceleration of the rate of joint space narrowing. Therefore, surgeons should pay attention to the fact that more than four interbody fusions worsen joint space narrowing of the hip.

## Data Availability

The datasets used and/or analyzed during the current study are available from the corresponding author on reasonable request.

## Abbreviations

ASD: Adjacent segmental degeneration
OA: Osteoarthritis
THA: Total hip arthroplasty
SVA: Sagittal vertical axis
TK: Thoracic kyphosis
LL: Lumbar lordosis
SS: Sacral slope
PT: Pelvic tilt
PI: Pelvic incidence
CE: Center edge
MJW: Minimum joint width
